# Incremental cardiovascular costs and resource use associated with diabetes: an assessment of 29,863 patients in the US managed-care setting

**DOI:** 10.1186/1475-2840-8-53

**Published:** 2009-09-26

**Authors:** Robert J Straka, Larry Z Liu, Prafulla S Girase, Allyson DeLorenzo, Richard H Chapman

**Affiliations:** 1 Department of Experimental and Clinical Pharmacology, University of Minnesota College of Pharmacy, Minneapolis, MN, USA; 2 Global Outcomes Research, Pfizer Inc, New York, NY and Weill Cornell Medical College, New York, NY, USA; 3 IMS Health, Watertown, MA, USA; 4 Columbia University, New York, NY, USA; 5 US Health Economics and Outcomes Research, IMS Health, Falls Church, VA, USA

## Abstract

**Background:**

Patients with type 2 diabetes are at increased risk of cardiovascular events, and there is an associated economic burden attached to this risk. We conducted a retrospective claims database analysis to evaluate incremental cardiovascular costs in diabetic versus non-diabetic patients hospitalized for a cardiovascular event.

**Methods:**

Patients hospitalized for a cardiovascular event between January 1, 2001 and June 30, 2005 were identified from a large US managed-care population. Diabetic patients were identified by evidence of type 2 diabetes in the 12 months prior to the index hospitalization. Direct medical costs and resource use - including inpatient expenditures (for the index and first recurrent hospitalizations), as well as outpatient, laboratory, and pharmacy expenditures (during the 3-year follow-up period) - were determined for patients with or without diabetes.

**Results:**

Of the 29,863 patients identified with a cardiovascular hospitalization, 5,501 patients (18.4%) had a history of diabetes in the pre-index period (mean age, 57.8 years; 42.1% female). The overall mean follow-up period was 22.8 months. The incidence of subsequent cardiovascular events in the first year of follow-up was significantly higher for patients with diabetes compared with non-diabetic patients for all types of cardiovascular events except angina. Compared with non-diabetic patients, patients with diabetes had similar mean direct medical costs per patient for the index cardiovascular hospitalization ($17,435 versus $16,917; *P *= 0.09), and the first recurrent cardiovascular hospitalization ($18,488 versus $17,481; *P *= 0.2), yet higher mean total direct medical costs per patient for cardiovascular events during follow-up years (Year 1: $8,805 versus $6,982; Year 2: $13,860 versus $10,056; Year 3: $16,149 versus $12,163; all *P *≤ 0.0002). The cost difference between diabetic and non-diabetic patients remained significant after adjusting for age, gender and other potential confounders in multivariate regression analysis. The mean (SD) total period of inpatient cardiovascular hospitalization after 3 years of follow-up was 3.3 (12.4) days for patients with diabetes compared with 1.8 (5.8) days for non-diabetic patients (*P *< 0.0001).

**Conclusion:**

Diabetic patients hospitalized for a cardiovascular event incur higher costs for cardiovascular care than their non-diabetic counterparts. This analysis of the incremental cardiovascular cost and resource use provides the basis for greater accuracy and precision when modeling the economic value of initiatives aimed at reducing cardiovascular morbidity in patients with diabetes mellitus.

## Introduction

Approximately 17.5 million people in the United States have recognized diabetes, with an estimated 1 million new cases diagnosed each year [1]. Diabetes is a well-established risk factor for future cardiovascular events [2], including coronary heart disease (CHD), ischemic stroke, and peripheral vascular disease. Of the ~284,000 deaths in 2007 attributed to diabetes and its associated complications, nearly 65% were due to cardiovascular or cerebrovascular causes [1]. Indeed, the presence of diabetes can increase the risk of CHD and stroke by as much as 5-fold [3-6], in addition to conferring a worse prognosis for survival from a cardiovascular event [7-10]. As such, diabetes is currently regarded as a CHD risk equivalent [11,12] and recommendations for aggressive risk factor management in the diabetic population reflect this high-risk status [13].

Given the prevalence of diabetes and the morbidity and mortality associated with it, the burden the disease imposes on the US economy is considerable: the total cost of diabetes in 2007 was estimated at $116 billion in medical expenditures and an additional $58 billion in lost productivity [1]. Patient-level estimates of the cost of cardiovascular care in diabetic populations are critical for pharmacoeconomic modeling and guiding decisions on disease management in this cohort. Although previous studies have investigated the impact of cardiovascular disease (CVD) on health-care costs for diabetes [14-20], few contemporary analyses have determined incremental cardiovascular costs among diabetic versus non-diabetic patients with pre-existing CVD, particularly those costs associated with specific types of cardiovascular events such as coronary artery bypass graft (CABG) procedures, myocardial infarction (MI), and ischemic stroke.

Therefore, using claims data representative of a large US managed-care population, we assessed the direct medical costs and resource use associated with initial and subsequent cardiovascular episodes in patients with or without diabetes hospitalized for a cardiovascular event.

## Methods

### Data source and claims

This retrospective cohort analysis assessed transactional billing records from the PharMetrics Patient-Centric Database, which contains fully adjudicated medical and pharmaceutical claims for > 50 million unique patients from > 90 health-care plans across the United States.

The database includes both inpatient and outpatient diagnoses (based on International Classification of Diseases, 9th Revision, Clinical Modification [ICD-9-CM] codes) and procedures (based on Current Procedural Terminology 4 [CPT-4] and Health Care Financing Agency Common Procedure Coding System [HCPCS] codes), in addition to both retail and mail-order prescription records (including Generic Product Identifier [GPI] codes). Both paid and charged amounts are available for all services rendered, as well as dates of service for all claims. Additional data elements include demographic variables (eg, age, gender, geographic region), product type (eg, Health Maintenance Organization, Preferred Provider Organization), payor type (eg, commercial, self-pay), provider specialty, and start and stop dates for plan enrollment. Only health-care plans that submit data for all plan members are included in the database, ensuring complete data capture and representative samples. Contributions are also subjected to a series of data quality checks to ensure a standardized format and minimal error rates.

### Study period

The overall study period was from January 1, 2001 to June 30, 2006. The patient identification period was from January 1, 2001 to June 30, 2005. The first hospitalization with relevant diagnosis or procedure codes for a cardiovascular event during the identification period was defined as the index hospitalization. The pre-index period was defined as the 12 months prior to the index hospitalization admission date. The post-index (follow-up) period began 1 day after the discharge date of index hospitalization. The follow-up period was stratified to define 3 cohorts of patients with 1, 2, or 3 years of continuous enrollment after the index hospitalization discharge date. Follow-up terminated at the earliest of patient disenrollment or June 30, 2006. Patients who died during the study were included in the analysis regardless of length of follow-up.

### Identification of study cohorts

**Figure 1:**
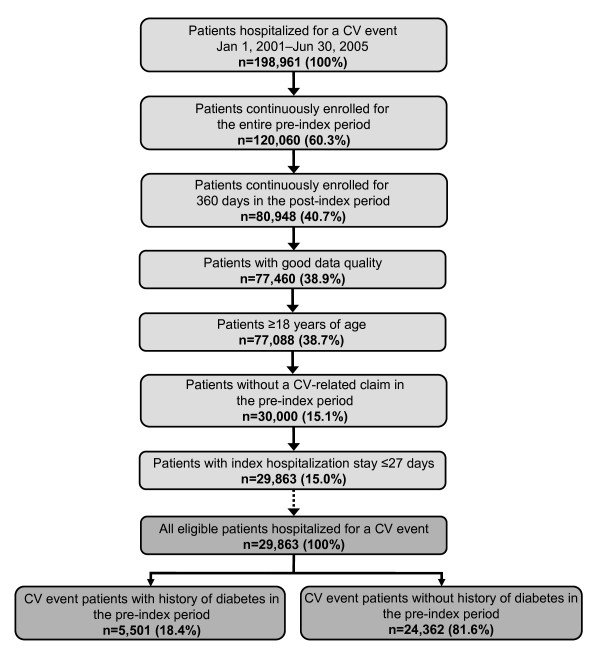
**The study cohort identification procedure with the final study cohort stratified by history of type 2 diabetes in the 12 months prior to hospitalization. CV, cardiovascular**. Figure provides an overview of the patient identification procedure with reasons for inclusion/exclusion through the selection process.

The patient selection procedure is shown in Figure 1. Patients with a complete hospitalization (admission and live discharge within the identification period) for a cardiovascular event between January 1, 2001 and June 30, 2005 were identified from the claims database on the basis of ICD-9-CM diagnosis codes and CPT-4 procedure codes (see Additional File 1). Inclusion in the final study cohort required patients to be continuously enrolled for the entire pre-index and follow-up periods, to not have a cardiovascular-related claim in the pre-index period, to have an index hospitalization stay ≤ 27 days, and to be ≥ 18 years of age at index hospitalization. Diabetic patients were identified by evidence of type 2 diabetes in the pre-index period (diagnosis of type 2 diabetes or antidiabetic medication use; Additional file 1).

### Cost analyses and resource utilization

All medical, laboratory, and pharmacy claims were assessed for the identified patients for the period from January 1, 2000 to June 30, 2006. Direct medical costs included any cost incurred during an inpatient hospitalization associated with a cardiovascular event. If a cardiovascular event was the primary diagnosis in any hospitalization (index or readmission), all costs from that hospitalization were attributed to that cardiovascular event and used in the calculation of costs. Costs were summed from the admission date to the discharge date for the hospitalization. Mean per patient costs were calculated for direct medical costs associated with the index cardiovascular event, direct medical costs associated with the first recurrent cardiovascular event, and total direct medical costs for cardiovascular events during the follow-up period. The mean hospital length of stay (LOS) associated with each cardiovascular event was also determined. Direct medical costs and resource utilization were calculated for all cardiovascular event types combined as well as stratified by specific cardiovascular event types (eg, CABG, MI, ischemic stroke); total direct medical costs during the follow-up period were also stratified by type of service (eg, inpatient, outpatient, pharmacy). Costs are reported in US dollars adjusted for inflation to 2006 using the medical care component of the Consumer Price Index.

Subsequent cardiovascular events following discharge from the index hospitalization were assessed by identifying complete hospitalizations during the follow-up period with the relevant diagnosis or procedure codes for a cardiovascular event (Additional file 1) to calculate the incidence of subsequent cardiovascular events during the first year of follow-up.

### Statistical methods

Mean and SD were calculated for continuous variables; frequency was calculated for categorical variables. Continuous outcome variables were compared using a *t*-test of means, and categorical outcome variables were compared using a Chi Square test. Multivariable regression analysis was performed using generalized linear models (GLM) with total cardiovascular-related costs after 1-year follow-up as the dependent variable, adjusting for age, gender, geographic region, insurance type, physician specialty and relevant co-morbidities, including diabetes status. Statistical analyses were conducted using the SPSS^® ^(SPSS Inc, Chicago, IL) and SAS^® ^(SAS Institute Inc, Cary, NC) software suites.

## Results

### Study cohorts

A total of 198,961 patients were identified as having a hospitalization for a cardiovascular event between January 1, 2001 and June 30, 2005 (Figure 1); of these, 29,863 patients were eligible for inclusion in the final study cohort. Exclusion from the study was due primarily to the requirement of continuous enrollment for the entire pre-index period and for 360 days in the post-index period, and to not have a cardiovascular-related claim in the pre-index period. Within this overall study population, 5,501 patients (18.4%) were identified as having a history of type 2 diabetes in the 12 months prior to hospitalization (Figure 1).

### Baseline demographics and clinical characteristics

The baseline demographics and clinical characteristics of the study cohorts are shown in Table 1. Overall, the patient population primarily consisted of commercially insured patients belonging to either a Health Maintenance Organization or Preferred Provider Organization health-care plan. The mean (SD) follow-up period was 22.8 months. Compared with non-diabetic patients and the study population overall, the cohort of patients with diabetes was slightly older, comprised more women, and had a higher incidence of comorbidity on the basis of the Charlson Comorbidity Index score and prevalence of hypertension and dyslipidemia (Table 1). As such, patients with diabetes were more likely to be taking lipid-lowering and antihypertensive medications. The cardiovascular event type at index hospitalization was similar across the study cohorts, with the exception of MI (lower incidence in diabetic patients) and heart failure (higher incidence in diabetic patients).

**Table 1:** Baseline demographics and clinical characteristics of the study cohort overall and by diabetic status

**Parameter**	**All Patients** **(n = 29,863)**	Patients with Diabetes* (n = 5,501)	Patients without Diabetes* (n = 24,362)	***P*-value^‡^**
Age, mean (SD), years	55.54 (12.05)	57.83 (11.00)	55.03 (12.22)	< 0.0001
Female, n (%)	11,097 (37.2)	2,316 (42.1)	8,781 (36.0)	< 0.0001
Follow-up, mean (SD), days	694 (348)	687 (353)	695 (347)	0.12
Plan type, n (%)				< 0.0001
HMO	12,167 (40.7)	2,444 (44.4)	9,723 (39.9)	
PPO	11,356 (38.0)	1,941 (35.3)	9,415 (38.6)	
Unknown	306 (1.0)	48 (0.9)	258 (1.1)	
Plan payer type, n (%)				< 0.0001
Commercial	22,270 (74.6)	3,841 (69.8)	18,429 (75.6)	
Medicare risk	4,199 (14.1)	974 (17.7)	3,225 (13.2)	
Unknown	1,587 (5.3)	222 (4.0)	1,365 (5.6)	
CCI, mean (SD)	0.56 (1.12)	1.61 (1.22)	0.32 (0.94)	< 0.0001
Pre-index comorbidities, n (%)				
Hypertension	11,648 (39.0)	3,408 (62.0)	8,240 (33.8)	< 0.0001
Dyslipidemia	8,976 (30.1)	2,642 (48.0)	6,334 (26.0)	< 0.0001
Diabetes	5,501 (18.4)	5,501 (100.0)	0 (0.0)	-
Pre-index medications, n (%)				
Statins	4,749 (15.9)	1,708 (31.0)	3,041 (12.5)	< 0.0001
ACE inhibitors	4,027 (13.5)	1,805 (32.8)	2,222 (9.1)	< 0.0001
ARBs	1,316 (4.4)	473 (8.6)	843 (3.5)	< 0.0001
β-blockers	4,045 (13.5)	1,086 (19.7)	2,959 (12.1)	< 0.0001
Calcium channel blockers	3,070 (10.3)	970 (17.6)	2,100 (8.6)	< 0.0001
Diuretics	2,919 (9.8)	849 (15.4)	2,070 (8.5)	< 0.0001
Index CV event type^†^				
CABG	1,904 (6.4)	412 (7.5)	1,492 (6.1)	0.0002
Other CV procedure	8,613 (28.8)	1,417 (25.8)	7,196 (29.5)	< 0.0001
Myocardial infarction	9,231 (30.9)	1,420 (25.8)	7,811 (32.1)	< 0.0001
Heart failure	2,244 (7.5)	686 (12.5)	1,558 (6.4)	< 0.0001
Angina	1,651 (5.5)	315 (5.7)	1,336 (5.5)	0.48
Other IHD	11,258 (37.7)	2,011 (36.6)	9,247 (38.0)	0.053
Ischemic stroke	2,634 (8.8)	552 (10.0)	2,082 (8.5)	0.0004
TIA/other CVA	2,324 (7.8)	395 (7.2)	1,929 (7.9)	0.07
PVD	164 (0.5)	38 (0.7)	126 (0.5)	0.12

ACE, angiotensin-converting enzyme; ARB, angiotensin II receptor blocker; CABG, coronary artery bypass graft; CCI, Charlson Comorbidity Index; CV, cardiovascular; CVA, cerebrovascular accident; HMO, Health Maintenance Organization; IHD, ischemic heart disease; PPO, Preferred Provider Organization; PVD, peripheral vascular disease; TIA, transient ischemic attack.

*History of diabetes in the pre-index period. ^†^Index hospitalization may be categorized into multiple events if it had both a cardiovascular diagnosis and procedure. ^‡^*P*-values for comparison of patients with vs. without diabetes; χ^2 ^test for categorical variables, and t-test for continuous variables

### Impact of diabetes on incidence of subsequent cardiovascular events

The proportion of patients in each study cohort experiencing a subsequent cardiovascular event during the first year of follow-up is shown in Table 2. Patients with diabetes had a higher incidence of each specific cardiovascular event at 1 year of follow-up than patients without diabetes (Table 2). The incidence of subsequent cardiovascular events in the first year of follow-up was significantly higher for patients with diabetes compared with non-diabetic patients for all types of cardiovascular event excluding angina (all *P *≤ 0.03).

**Table 2:** Incidence of subsequent cardiovascular events in 1 year of follow-up by subsequent cardiovascular event type and diabetic status

Subsequent CV Event Type*	**Patients with Diabetes^†‡^** **(n = 5,501)**	**Patients without Diabetes^†‡^** **(n = 24,362)**	**P-value** **(χ^2 ^test)**
CABG	147 (2.7)	425 (1.7)	< 0.001
Other CV procedure	391 (7.1)	1,395 (5.7)	< 0.001
Myocardial infarction	129 (2.3)	375 (1.5)	< 0.001
Heart failure	197 (3.6)	425 (1.7)	< 0.001
Angina	23 (0.4)	83 (0.3)	0.38
Other IHD	613 (11.1)	2,454 (10.1)	0.02
Ischemic stroke	105 (1.9)	300 (1.2)	< 0.001
TIA/other CVA	76 (1.4)	212 (0.9)	< 0.001
PVD	29 (0.5)	80 (0.3)	0.03

CABG, coronary artery bypass graft; CV, cardiovascular; CVA, cerebrovascular accident; IHD, ischemic heart disease; PVD, peripheral vascular disease; TIA, transient ischemic attack.

*Subsequent cardiovascular event may be categorized into multiple events if it had both a cardiovascular diagnosis and procedure.

^†^History of diabetes in the pre-index period. ^‡^All values are n (%).

*P*-values for comparison of patients with vs. without diabetes

### Impact of diabetes on health-care costs

In the 12 months prior to the index cardiovascular hospitalization, patients with diabetes incurred higher total health-care costs per patient (mean [SD]: $8,394 [$18,549]) compared with non-diabetic patients ($4,405 [$12,037]; *P *< 0.0001).

Diabetic patients had similar direct medical costs per patient for the index cardiovascular hospitalization (mean [SD]: $17,435 [$21,613]) than patients without diabetes ($16,917 [$20,342]; *P *= 0.09) (Table 3). An analysis of the specific type of cardiovascular event underlying the index hospitalization showed that, compared with those without diabetes, patients with diabetes had higher costs for those hospitalizations associated with CABG procedures, MI, angina, other ischemic heart disease (e.g. coronary atherosclerosis), and peripheral vascular disease (Table 3). For both study cohorts, the highest index hospitalization costs were incurred for hospitalizations related to CABG procedures (diabetics: $60,385 [$38,622]; non-diabetics: $55,004 [$32,109]), followed by hospitalizations for MI and other cardiovascular procedures (eg, non-CABG revascularizations) (Table 3).

**Table 3:** Mean direct medical costs and length of stay for the index cardiovascular hospitalization by index cardiovascular event type and diabetic status

Index CV Event Type*	**Patients with Diabetes^†^** **(n = 5,501)**			**Patients without Diabetes^†^** **(n = 24,362)**		
	
	**No. of Patients**	**Cost of Hospitalization^‡^**	**Hospital LOS^§^**	**No. of Patients**	**Cost of Hospitalization^‡^**	**Hospital LOS^§^**
All CV event types**	5,501	17,435 (21,613)	4.8 (3.5)	24,362	16,917 (20,342)	4.3 (3.0)
CABG	412	60,385 (38,622)	10.0 (3.9)	1,492	55,004 (32,109)	9.0 (3.6)
Other CV procedure	1,417	25,925 (19,369)	4.1 (2.4)	7,196	26,331 (21,446)	4.1 (2.4)
Myocardial infarction	1,420	26,035 (27,918)	5.2 (3.6)	7,811	24,802 (24,278)	4.6 (2.9)
Heart failure	686	11,178 (13,168)	5.7 (3.7)	1,558	12,560 (16,245)	5.6 (3.6)
Angina	315	7,038 (6,975)	2.9 (1.4)	1,336	6,878 (7,722)	2.7 (1.4)
Other IHD	2,011	17,869 (20,538)	4.2 (3.0)	9,247	14,740 (17,549)	3.7 (2.6)
Ischemic stroke	552	11,802 (12,025)	5.6 (3.7)	2,082	12,667 (13,344)	5.4 (3.3)
TIA/other CVA	395	6,638 (6,733)	4.0 (3.4)	1,929	6,711 (6,379)	3.7 (2.7)
PVD	38	19,432 (25,386)	8.1 (6.4)	126	15,315 (17,835)	6.6 (5.0)

CABG, coronary artery bypass graft; CV, cardiovascular; CVA, cerebrovascular accident; IHD, ischemic heart disease; LOS, length of stay; PVD, peripheral vascular disease; TIA, transient ischemic attack.

*Index hospitalization may be categorized into multiple events if it had both a cardiovascular diagnosis and procedure. ^†^History of diabetes in the pre-index period. ^‡^Costs are mean (SD) per patient in US$. ^§^Length of stay is mean (SD) in days. **For *t*-test comparisons of all CV event patients with vs. without diabetes, cost of hospitalization: *P *= 0.09, hospital length of stay: *P *< 0.0001

For the first recurrent cardiovascular hospitalization, mean (SD) direct medical costs per patient were $18,488 ($22,014) for patients with diabetes, compared with $17,481 ($22,895) for non-diabetics (*P *= 0.2) (Table 4). Diabetic patients had higher costs for recurrent hospitalizations associated with other cardiovascular procedures, MI, other ischemic heart disease, ischemic stroke, and peripheral vascular disease (Table 4). As with the index hospitalization, the highest recurrent hospitalization costs for all study cohorts were for hospitalizations related to CABG procedures (diabetics: $43,404 [$28,686]; non-diabetics: $47,110 [$39,612]) (Table 4).

**Table 4:** Mean direct medical costs and length of stay for the first recurrent cardiovascular hospitalization by first Recurrent cardiovascular event type and diabetic status

First Recurrent CV Event Type*	**Patients with Diabetes^†^** **(n = 5,501)**			**Patients without Diabetes^†^** **(n = 24,362)**		
	
	**No. of Patients**	**Cost of Hospitalization^‡^**	**Hospital LOS^§^**	**No. of Patients**	**Cost of Hospitalization^‡^**	**Hospital LOS^§^**
All CV event types**	1,071	18,488 (22,014)	5.6 (12.6)	3,734	17,481 (22,895)	4.6 (7.3)
CABG	112	43,404 (28,686)	10.8 (33.6)	338	47,110 (39,612)	8.3 (7.1)
Other CV procedure	346	19,831 (14,867)	3.2 (2.3)	1,281	18,807 (15,588)	2.9 (2.6)
Myocardial infarction	106	22,854 (33,721)	8.2 (34.8)	332	22,125 (27,934)	5.1 (5.0)
Heart failure	168	14,031 (21,581)	7.5 (6.9)	363	14,398 (26,197)	5.7 (5.4)
Angina	18	8,016 (9,243)	3.9 (4.0)	72	8,477 (9,039)	3.2 (2.3)
Other IHD	554	21,445 (21,768)	4.3 (5.5)	2,316	18,038 (19,859)	3.6 (3.2)
Ischemic stroke	96	11,168 (8,687)	5.0 (4.0)	267	10,804 (10,594)	6.0 (8.3)
TIA/other CVA	64	7,501 (6,720)	10.0 (14.1)	179	9,195 (11,887)	12.8 (24.6)
PVD	25	23,146 (23,278)	7.6 (5.3)	61	14,788 (13,850)	4.9 (4.4)

CABG, coronary artery bypass graft; CV, cardiovascular; CVA, cerebrovascular accident; IHD, ischemic heart disease; LOS, length of stay; PVD, peripheral vascular disease; TIA, transient ischemic attack.

*First recurrent hospitalization may be categorized into multiple events if it had both a cardiovascular diagnosis and procedure. ^†^History of diabetes in the pre-index period. ^‡^Costs are mean (SD) per patient in US$. ^§^Length of stay is mean (SD) in days. **For *t*-test comparisons of all CV event patients with vs. without diabetes, cost of hospitalization: *P *= 0.20, hospital length of stay: *P *= 0.0008.

**Figure 2:**
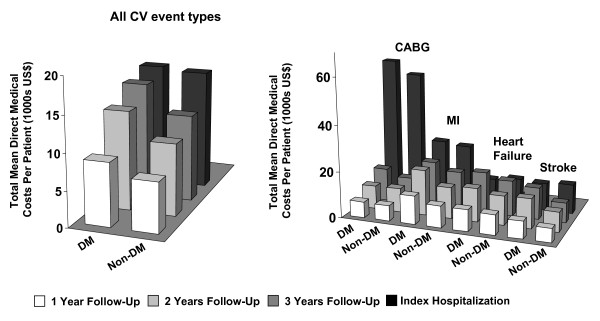
**Total mean direct medical costs per patient over 3 years of follow up for all cardiovascular events (left-hand graph) and for selected cardiovascular event types (right-hand graph)**. CABG, coronary artery bypass graft; CV, cardiovascular; DM, diabetes mellitus; MI, myocardial infarction. Data provided represent the mean total cumulative direct medical costs per patient for cardiovascular events across 3 years of follow-up for all cardiovascular events and for selected event types.

Mean total cumulative direct medical costs per patient for cardiovascular events across 3 years of follow-up are shown in Table 5 and Figure 2. With the exception of costs associated with CABG procedures in the second year of follow-up, diabetic patients incurred higher costs for follow-up care for all cardiovascular event types assessed than patients without diabetes (Table 5). The highest follow-up costs for both study cohorts were incurred for MI and other cardiovascular procedures. Follow-up costs were generally driven by inpatient hospitalization costs (LOS) as well as costs associated with outpatient care (eg, physician office visits, imaging tests), particularly in diabetic patients. Cardiovascular-related pharmacy costs (mean [SD]) during the follow-up period were also higher for diabetic patients (Year 1: $964 [$1,060]; Year 2: $2,049 [$2,007]; Year 3: $3,013 [$2,910]) compared with non-diabetic patients (Year 1: $774 [$1,044]; Year 2: $1,588 [$1,844]; Year 3: $2,406 [$2,477]).

**Table 5:** Cumulative total direct medical costs and length of stay for cardiovascular events during 3 years of follow-up by follow-up cardiovascular event type and diabetic status

Follow-up CV Event Type*	**Patients with Diabetes^†^** **(n = 5,501)**			**Patients without Diabetes^†^** **(n = 24,362)**		
	
	**No. of Patients**	**Cost of** **Follow-Up^‡^**	**Hospital LOS^§^**	**No. of Patients**	**Cost of** **Follow-Up^‡^**	**Hospital LOS^§^**
**1 Year**:						
All patients with CV events**	5,501	8,805 (22,277)	1.6 (7.4)	24,362	6,982 (18,545)	1.0 (4.8)
CABG	412	6,894 (13,122)	0.7 (2.8)	1,492	6,690 (13,159)	0.6 (2.8)
Other CV procedure	1,417	11,195 (19,917)	1.3 (3.7)	7,196	9,628 (22,946)	0.9 (3.5)
Myocardial infarction	1,420	12,256 (31,941)	1.7 (5.2)	7,811	9,502 (24,244)	1.1 (4.4)
Heart failure	686	9,324 (22,806)	3.3 (16.4)	1,558	8,711 (26,334)	1.9 (6.4)
Angina	315	5,463 (19,684)	0.6 (3.1)	1,336	3,985 (11,352)	0.4 (1.6)
Other IHD	2,011	8,053 (17,264)	1.2 (4.4)	9,247	6,167 (14,279)	0.8 (3.4)
Ischemic stroke	552	7,521 (13,388)	2.2 (7.0)	2,082	6,052 (11,827)	1.9 (9.4)
TIA/other CVA	395	3,648 (8,469)	0.9 (3.6)	1,929	2,544 (9,369)	0.6 (5.3)
PVD	38	8,537 (20,215)	2.5 (5.0)	126	6,163 (17,706)	0.9 (3.2)

**2 Years**:						
All patients with CV events^††^	2,132	13,860 (26,619)	2.3 (8.6)	9,522	10,056 (22,677)	1.3 (5.3)
CABG	155	10,161 (14,314)	2.3 (19.6)	589	10,313 (17,319)	0.9 (3.8)
Other CV procedure	560	17,980 (29,350)	2.5 (11.5)	2,806	13,980 (24,577)	1.2 (3.8)
Myocardial infarction	530	19,703 (37,992)	2.9 (12.1)	3,091	13,520 (27,764)	1.4 (5.1)
Heart failure	251	14,535 (27,065)	4.1 (11.6)	568	12,606 (35,669)	2.6 (8.2)
Angina	129	6,869 (13,410)	1.2 (4.0)	576	5,015 (11,391)	0.4 (1.7)
Other IHD	802	12,485 (20,869)	1.6 (4.4)	3,585	9,207 (18,789)	1.1 (3.8)
Ischemic stroke	211	12,893 (19,845)	3.4 (9.5)	694	8,686 (15,325)	1.9 (8.2)
TIA/other CVA	155	6,916 (14,687)	1.5 (7.3)	844	4,576 (15,012)	1.0 (7.8)
PVD	14	9,802 (14,433)	1.1 (3.3)	52	5,380 (10,697)	0.7 (2.0)

**3 Years**:						
All patients with CV events^‡‡^	735	16,149 (27,290)	3.3 (12.4)	3,141	12,163 (26,268)	1.8 (5.8)
CABG	54	14,299 (20,342)	5.6 (34.4)	189	11,445 (17,920)	1.1 (3.9)
Other CV procedure	174	21,821 (29,208)	4.2 (19.7)	864	17,150 (24,413)	1.5 (3.7)
Myocardial infarction	194	19,773 (32,653)	4.0 (18.9)	984	16,566 (33,829)	2.0 (6.0)
Heart failure	84	17,641 (33,665)	6.2 (17.5)	198	15,309 (40,834)	2.9 (7.0)
Angina	45	10,744 (19,220)	1.9 (6.6)	185	4,844 (9,124)	0.5 (1.5)
Other IHD	268	16,428 (25,001)	2.7 (5.8)	1,179	12,031 (21,739)	1.9 (6.3)
Ischemic stroke	71	13,654 (19,163)	3.3 (8.9)	242	8,526 (14,211)	1.8 (6.0)
TIA/other CVA	62	9,045 (21,836)	1.4 (4.2)	284	4,400 (10,622)	0.8 (3.8)
PVD	2	10,119 (10,258)	0.0 (0.0)	22	6,678 (14,186)	1.2 (2.6)

CABG, coronary artery bypass graft; CV, cardiovascular; CVA, cerebrovascular accident; IHD, ischemic heart disease; LOS, length of stay; PVD, peripheral vascular disease; TIA, transient ischemic attack.

*Follow-up cardiovascular event may be categorized into multiple events if it had both a cardiovascular diagnosis and procedure. ^†^History of diabetes in the pre-index period. ^‡^Costs are incremental to that of the index cardiovascular hospitalization and given as mean (SD) per patient in US$. ^§^Length of stay is mean (SD) in days.

**For t-test comparisons of all CV event patients with vs. without diabetes after 1 year cost of hospitalization: *P *< 0.0001, hospital length of stay: *P *< 0.0001; ^††^after 2 years, cost of hospitalization: *P *< 0.0001, hospital length of stay: *P *< 0.0001; ^‡‡^after 3 years, cost of hospitalization: *P *= 0.0002, hospital length of stay: *P *< 0.0001.

In a multivariable GLM analysis adjusting for age, gender and other patient characteristics, total cardiovascular-related costs after 1-year follow-up were more likely to be higher for patients with diabetes vs. those without diabetes (adjusted relative risk = 1.28, 95% confidence interval = 1.22-1.35, p < 0.0001).

### Impact of diabetes on health-care resource use

Patients with diabetes had a mean (SD) LOS for the index hospitalization of 4.8 (3.5) days compared with 4.3 (3.0) days for non-diabetic patients (*P *< 0.0001) (Table 3). For both study cohorts, the longest periods of index hospitalization were associated with CABG procedures (diabetics: 10.0 [3.9] days; non-diabetics: 9.0 [3.6] days), followed by hospitalizations with a diagnosis of peripheral vascular disease (diabetics: 8.1 [6.4] days; non-diabetics: 6.6 [5.0] days) (Table 3).

For the first recurrent cardiovascular hospitalization, mean (SD) LOS was 5.6 (12.6) days for patients with diabetes, compared with 4.6 (7.3) days for non-diabetics (*P *= 0.0008) (Table 4). Recurrent hospitalizations with the longest periods of inpatient stay for both study cohorts were those with a diagnosis of transient ischemic attack or other cerebrovascular accident and those involving CABG procedures (Table 4).

Mean LOS for cardiovascular hospitalizations across 3 years of follow-up are shown in Table 5. With the exception of inpatient stays with a diagnosis of peripheral vascular disease in the third year of follow-up, where data on only 2 patients with diabetes were available, diabetic patients had longer periods of follow-up hospitalization for all cardiovascular event types assessed than patients without diabetes (Table 5). The longest periods of follow-up hospitalization for both study cohorts were those associated with a diagnosis of heart failure (Table 5). Overall, the total period of inpatient cardiovascular hospitalization after 3 years of follow-up was 3.3 (12.4) days for patients with diabetes compared with 1.8 (5.8) days for non-diabetic patients (*P *< 0.0001).

## Discussion

In this retrospective claims database analysis, we provide quantitative documentation of the differential costs associated with managing patients with diabetes hospitalized for a cardiovascular event within US managed-care settings. Our observations that the diabetic population incurs higher direct medical costs for cardiovascular care during the initial hospitalization and follow-up period than their non-diabetic counterparts is not new; however, in light of advances in medical care and the pertinence of its setting (within a US managed care population), this contemporary assessment is relevant. We confirm that patients with diabetes also experience longer periods of inpatient cardiovascular hospitalization than those without diabetes. The higher costs and resource use in the diabetic cohort likely reflect the combination of a higher incidence of subsequent cardiovascular events observed in this population compared with patients without diabetes as well as a higher overall comorbidity index. In addition to evaluating the incremental costs and resource use associated with cardiovascular events overall, this study is the first of its kind to provide an economic assessment of this nature for specific cardiovascular event types such as CABG procedures, MI, and ischemic stroke.

Excess medical costs for the initial cardiovascular hospitalization in the diabetic cohort were primarily driven by large cost differentials for those hospitalizations associated with CABG procedures, other ischemic heart disease (eg, coronary atherosclerosis), and peripheral vascular disease - CVD types commonly associated with diabetes.

During the follow-up period, diabetic patients experienced a higher incidence of subsequent cardiovascular events than the non-diabetic cohort for each event type (Table 2). As such, medical costs for cardiovascular care during the follow-up period were consistently higher in the diabetic cohort. All cardiovascular event types examined in this analysis contributed to excess follow-up costs in patients with diabetes, with cost differentials between the diabetic and non-diabetic cohorts increasing with each successive year of follow-up. Inpatient hospitalization costs and costs associated with outpatient care, such as physician office visits and imaging tests, were the primary drivers of follow-up cardiovascular costs irrespective of diabetic status.

Taking into account expenditures for the initial cardiovascular hospitalization plus all cardiovascular events during the follow-up period, the incremental per-patient cost attached to cardiovascular events in patients with diabetes was $10,131 over 3 years. Extrapolation of this additional cost across the ~5,500 diabetic patients identified from this health-care plan reveals that the 3-year excess cardiovascular costs for this population totaled nearly $56 million, or > $18.5 million per year.

The majority of studies that have investigated the impact of comorbid conditions on health-care expenditure for diabetes have found that cardiovascular complications are the most costly component of medical care in this population [14-16,19,20]. For example, a recent model of the lifetime costs of complications associated with diabetes found that macrovascular disease accounted for 85% of cumulative costs in the first 5 years and 52% of costs over 30 years [19].

Furthermore, we and others [14,15,18] have shown that the cost of CVD is notably higher in diabetic patients compared with similar patients without diabetes, likely due to the severity of cardiovascular events experienced by diabetic individuals as indicated by a worse prognosis for survival [7-10]. A recent study [18] assessing annual medical-care costs in diabetic patients from a large managed-care organization found that patients with both diabetes and CVD had higher medical-care costs than non-diabetic patients with CVD ($10,172 versus $6,396 per patient per year). These estimates are somewhat lower than the costs obtained in our analysis, where annual costs per patient for follow-up cardiovascular events were up to $16,149 for diabetic patients and $12,163 for non-diabetic patients in the third year of follow-up. The higher costs reported in our analysis in part reflects the fact that costs are from 2006 (versus 1999 costs in the previous study). Also, our analysis is based on actual costs paid by health-care plan providers in the PharMetrics database, as opposed to applying standard unit costs to resource-use profiles in a not-for-profit health maintenance organization, which may have the effect of underestimating the cost of care of these patients relative to our setting. Another study estimating direct medical costs associated with an initial event and 1 year of follow-up care for specific cardiovascular complications in diabetic patients found that the total event cost was $30,364 for MI; $6,024 for angina; $40,209 for ischemic stroke; and $3,874 for TIA [20]. Although closer to our assessment of cardiovascular costs in diabetic patients, these costs are still generally lower than those obtained in our analysis, again likely due to the use of standard unit costs from the year 2000.

As a retrospective analysis of a health-care plan claims database, this study is not without limitations. The use of diagnosis, procedure, and medication codes to identify patients and assess downstream costs and resource use relies on the accurate assignment of these codes to patient records in order to faithfully capture an individual's medical history. Contributions to the database used in this analysis are subjected to data quality checks to ensure minimal error rates. Furthermore, only health-care plans that submitted data for all plan members are included in the database, ensuring complete data capture and representative samples. Another potential limitation of this study is that the analysis of direct medical costs focuses the interpretation of the results to a payor's perspective and to US managed-care populations. However, the records in this database are representative of the national, commercially-insured population on a variety of demographic measures including age, gender, and plan type. The data are also longitudinal, with an average member enrollment time of 2 years. Hence, the results of this analysis may be generalized to similar managed-care populations at a nation-wide level.

## Conclusion

The results of the current analysis demonstrate that the incidence of subsequent events in the first year following hospitalization for an initial cardiovascular episode is significantly higher for patients with diabetes compared with non-diabetic patients. As a consequence of this increased cardiovascular burden, diabetic patients hospitalized for a cardiovascular event incur higher costs for cardiovascular care than their non-diabetic counterparts. The real-world cost estimates described here will aid the development of future economic models that assess the impact of health-care initiatives aimed at this growing diabetic population.

## Competing interests

RJS and AD were paid consultants to Pfizer Inc in connection with the development of this manuscript. PSG and RHC are employees of IMS Health, who were paid consultants to Pfizer Inc in the development of this manuscript. LZL is an employee of Pfizer Inc with ownership of stock in Pfizer Inc.

## Authors' contributions

RJS participated in the analysis and interpretation of the data and was involved in the development of the manuscript. LZL was involved in the conception and design of the study and participated in the analysis of the data and the development of the manuscript. PSG contributed to the design of the study and was involved in statistical analysis and programming. AD was involved in the conception and co-ordination of the study and participated in the development of the manuscript. RHC contributed to the conception, design and co-ordination of the study, and participated in statistical analysis and programming, analysis and interpretation of the data, and development of the manuscript. All authors read and approved the final manuscript.

## Supplementary Material

Additional file 1 **Diagnosis codes, procedure codes, and medications used in the identification of the study cohorts**. The codes provided were used to identify those patients within the database that had a hospitalization for a cardiovascular event and a history of type 2 diabetes. Click here for file
