# Supplementary material for: Incremental cardiovascular costs and resource use associated with diabetes: an assessment of 29,863 patients in the US managed-care setting
Source: Cardiovasc Diabetol. 2009 Sep 26;8:53. doi: 10.1186/1475-2840-8-53 (PMC2762466; doi:10.1186/1475-2840-8-53)
Supplement: Additional file 1 — Diagnosis codes, procedure codes, and medications used in the identification of the study cohorts. The codes provided were used to identify those patients within the database that had a hospitalization for a cardiovascular event and a history of type 2 diabetes. [file 1475-2840-8-53-S1.DOC]

**Supplementary Table 1: Diagnosis codes, procedure codes, and medications used in the identification of the study cohorts**

| **Description** | **Code (Type of Code)** |
| --- | --- |
| Cardiovascular events: | |
| Heart failure (with or without chronic kidney disease) | 398.91, 402.01, 402.11, 402.91, 404.01, 404.03, 404.11, 404.13, 404.91, 404.93, 428.xx (ICD-9-CM) |
| Myocardial infarction | 410.xx, 412 (ICD-9-CM) |
| Angina (unstable and pectoris) | 411.1, 413.x (ICD-9-CM) |
| Other ischemic heart disease | 411.xx (except 411.1), 414.xx, 427.xx, V45.81, V45.82 (ICD-9-CM) |
| Ischemic stroke | 433.xx, 434.xx, 436, 437.0, 437.1, 438.xx, 997.02 (ICD-9-CM) |
| Transient ischemic attack/other cerebrovascular accidents | 435.x (ICD-9-CM) |
| Peripheral vascular disease | 440.0, 440.1, 440.2x, 443.xx (ICD-9-CM) |
| Chronic obstructive pulmonary disease | 491.xx, 492.x, 496 (ICD-9-CM) |
| Revascularization  Coronary artery bypass graft  Coronary stenting  Percutaneous transluminal coronary angioplasty/ thrombectomy/atherectomy  Percutaneous transluminal pulmonary artery balloon angioplasty | 33503–33545 (CPT-4)  92980, 92981 (CPT-4)  92973, 92982, 92984, 92995, 92996 (CPT-4)  92997, 92998 (CPT-4) |
| Endarterectomy  Carotid endarterectomy | 35301, 35390, 35901 (CPT-4) |
| History of type 2 diabetes: | |
| Diabetes mellitus type 2 (controlled or uncontrolled) | 250.x0, 250.x2, 250.x (ICD-9-CM) |
| Sulfonylureas  Glimepiride  Glipizide  Glyburide  Other Acetohexamide Chlorpropamide Tolazamide Tolbutamide | 27200027 (GPI)  2720003000 (GPI)  2720004000, 2720004010 (GPI)  27200010 (GPI)  27200020 (GPI)  27200050 (GPI)  27200060 (GPI) |
| Amino acid derivatives  Nateglinide | 272340 (GPI) |
| Biguanides  Metformin HCl | 272500 (GPI) |
| Meglitinide analogues  Repaglinide | 2728 (GPI) |
| Alpha-glucosidase inhibitors  Acarbose  Miglitol | 27500010 (GPI)  27500050 (GPI) |
| Insulin-sensitizing agents  Pioglitazone HCl  Rosiglitazone maleate | 27607050 (GPI)  27607060 (GPI) |
| Antidiabetic combinations  Glipizide–Metformin HCl  Glyburide–Metformin HCl  Rosiglitazone maleate–Glimepiride  Pioglitazone HCl–Metformin HCl  Rosiglitazone maleate–Metformin HCl | 2799700235 (GPI)  2799700240 (GPI)  279978 (GPI)  279980 (GPI)  279980 (GPI) |

CPT-4, Current Procedural Terminology 4; GPI, Generic Product Identifier; ICD-9-CM, International Classification of Diseases, 9th Revision, Clinical Modification.
